# Facial hair whorl location, behavior, and ocular temperature as a physiological stress indicator in young Pura Raza Española dressage horses

**DOI:** 10.3389/fvets.2026.1709706

**Published:** 2026-02-19

**Authors:** Mercedes Valera, Ana Encina, María José Sánchez-Guerrero, Arantxa Rodríguez-Sainz de los Terreros, Ester Bartolomé

**Affiliations:** 1 Dpto. Agronomía, Área de Producción Animal, Escuela Técnica Superior de Ingeniería Agronómica (ETSIA), Seville, Spain; 2 Real Asociación Nacional de Criadores de Caballos de Pura Raza Española (ANCCE), Seville, Spain

**Keywords:** acute stress, behavioral profiling, equine temperament, hair whorl pattern, infrared thermography

## Abstract

**Introduction:**

Behavioral and emotional reactivity assessment in sport horses is essential for optimizing their management and sports performance, particularly in equestrian disciplines such as Dressage. This study investigates, in Pura Raza Española (PRE) horses, the association between facial hair whorl (FHW) patterns, behavioral traits, and physiological stress responses using ocular temperature (OT) assessed with infrared thermography as, an objective, non-invasive physiological stress indicator.

**Methods:**

A total of 98 male PRE horses, aged 4–6 years old and all gray coated, were evaluated during the 2022–2024 Young Horse Selection Tests for Dressage. The FHW that were present on these PRE horses were classified by number and location (above, along, or below the eye line), while OT was recorded at rest, immediately post-competition, and several hours post-competition. Seven behavioral traits assessed via a standardized rider survey were also analyzed. Behavioral traits were assessed during the competition period through a standardized questionnaire completed by the regular rider, reflecting the horse’s general behavioral profile rather than responses to a single test.

**Results and discussion:**

Most horses presented a single FHW, predominantly along the eye line (60.2%). Behaviorally, a balanced profile was common, with strong correlations observed between aggression and dominance (*r* = 0.75, *p* < 0.001), and learning and cooperation (*r* = 0.72, *p* < 0.001). Eye temperature increased significantly just after competition, with higher values in horses with FHW along the eye line (*p* = 0.019). The number and location of FHW were associated with some interactions between nervousness, dominance, and/or aggression (*p* < 0.05). Multivariate analyses identified three distinct clusters, with the most favorable group characterized by FHW below the eye line, lower post-test temperatures, and high learning and cooperation scores. These findings demonstrate that, although FHW alone are not sufficient to predict temperament or stress reactivity, their integration with behavioral and physiological measures allows the empirical identification of distinct functional profiles in PRE horses.

## 1 Introduction

For sport horses, behavioral assessment is crucial for both routine management and selecting horses with the right temperament for competition. This assessment, which has traditionally relied on observational scales and questionnaires for riders and trainers (1), has progressively evolved towards more objective methodologies that incorporate physiological indicators to assess emotional response. Among these tools, infrared thermography (IRT) has been highlighted as a noninvasive and effective technique for studying the emotional state of equines (2, 3). Its ability to record subtle temperature changes in particularly sensitive regions, such as the eyes or the face, allows us to indirectly infer the activation of the autonomic nervous system during potentially stressful situations, such as transport, training, or participation in sporting events (4–7). In competitive contexts, Valera et al. (3) observed that ocular temperature levels increased significantly in young horses after participating in show jumping events, and that these thermal variations correlated with subjective indicators of stress. Furthermore, research carried out on Pura Raza Española (PRE) horses has shown that the ocular thermal response in competition situations presented significant interindividual variability, with an identifiable genetic basis, which reinforced its potential value as a tool in selection breeding programs for sports performance (8). In this sense, thermography not only provides information on animal welfare but could also facilitate the identification of individuals with a better capacity for emotional adaptation to the competitive environment.

In addition to its application as a diagnostic tool for stress, thermography has been used in research analyzing the correlation between physiological reactivity and certain personality or temperament traits (1, 9, 10). Although not all studies have used structured surveys, some studies suggest more anxious behaviors in horses showing higher eye temperature (11).

Furthermore, the possible relationship between external morphological characteristics and behavior has been explored in several livestock species. In particular, the location and number of facial hair whorls (FHW) have attracted interest as a potential phenotypic marker of temperament. Grandin et al. (12) observed in cattle that animals with FHW located above the eye line tended to show greater agitation during restraint, whereas those with lower FHW displayed a calmer demeanor. These findings suggested that FHW may reflect differences in emotional reactivity or in how animals cope with novel or challenging situations. Subsequent studies in cattle had extended this line of research by linking FHW location with behavioral patterns and with indirect indicators of health (13–15).

In horses, although studies were more limited, associations between FHW and behavior have also been reported by Merkies et al. (16) and Grandin and Deesing (17). Merkies et al. (16) noted that the position of FHW could be related to brain anatomy, specifically the location of the olfactory bulbs, which could have implications for sensory perception and the way stimuli were processed. This possible neuroanatomical link offered a biological basis for empirical observations by caregivers and trainers and opened a research avenue to evaluate FHW as a surrogate marker of temperament or emotional reactivity in equines. However, its practical utility as a predictor of behavior or the physiological response to stress has not yet been sufficiently explored, especially in equine populations selected for sports performance.

Beyond behavioral associations, embryological evidence has also been considered. From an embryological perspective, it had been proposed that FHW shared origins with the central nervous system, forming during very early stages of fetal development (17). This ontogenetic coincidence reinforced its potential as an external marker of internal processes related to brain lateralization, emotional processing, and reactivity. It should be emphasized that this proposed relationship is theoretical and based on embryological coincidence rather than on direct evidence of functional or anatomical connections between facial hair whorls and the brain or sensory systems.

The Pura Raza Española (PRE) horse represents a particularly suitable model for this type of integrative approach. Beyond its cultural relevance, the PRE has been subjected to long-term selection for behavioral stability, trainability, and functional performance in disciplines such as Dressage (18). Moreover, previous studies in this breed have documented genetic variability in both behavioral traits and physiological stress responses assessed through infrared thermography (8), providing a solid scientific background for exploring the association between morphological markers, temperament, and stress reactivity in a sport-oriented equine population.

The genetic basis of the FHW (19) and of the level of stress in equestrian competitions (8) have already been proven in the Pura Raza Española (PRE) horse breed, assessed in this study. The PRE is one of the most emblematic examples of Spain’s genetic, cultural, and sporting heritage. This breed is characterized by a harmonious morphology and a compact body, as well as a docile, noble, and balanced temperament that has favored its historical use in various equestrian disciplines that require great rapport with the rider, such as Dressage (8, 20). These qualities make it a functionally versatile and emotionally stable animal, with a good disposition for learning and work (21). In this type of discipline, not only physical aptitude but also behavior and emotional responses play a determining role in both athletic performance and animal welfare (22, 23). However, despite the importance of this breed, few studies have addressed the relationship between morphological variables (such as the location and number of FHW), physiological variables, and behavioral variables. Exploring these associations could provide new tools applicable to functional selection and individualized training of horses destined for sports.

Thus, the general objective of this study was to analyze the possible association between the location and number of FHW in young PRE horses, their behavioral characteristics, and their physiological response to stress, assessed using ocular IRT, during Dressage competitions. We hypothesized that horses with FHW located below the eye line would exhibit lower ocular temperature increases and more favorable behavioral profiles, defined by higher cooperation and learning ability together with lower levels of nervousness and excitability, compared to those with FHW located above the eye line.

## 2 Materials and methods

### 2.1 Animals

This study included 98 PRE horses participating in three final events of the Young Horse Selection Tests (YHST) for Dressage that were held at the Dos Lunas Polo Dressage Equestrian Center (Cádiz, Spain) between 2022 and 2024. The number and location of facial hair whorls were recorded, defined as hair growth patterns radiating from a central point (19). All participating horses were gray-coated males aged 4–6 years (29 horses were 4 years old, 33 were 5 years old, and 36 were 6 years old). Only male horses were included in the study to reduce heterogeneity related to sex. In addition, coat color was restricted to gray, the predominant coat in the PRE breed (24), to avoid introducing additional variability in facial hair whorl patterns associated with coat color, as previously reported in PRE horses (19). The study included all PRE horses that met the inclusion criteria during the final YHST events held between 2022 and 2024, namely participation in the Dressage finals, male sex, gray coat color, age between 4 and 6 years, and availability of complete facial hair whorl, thermographic, and behavioral data.

### 2.2 Young horse selection test (YHST)

The main objective of YHST is to contribute to the genetic improvement of equine breeds through the early evaluation of young offspring (aged 4–7 years old), based on performance monitoring and recording, with the goal of selecting future breeding animals. These evaluations prioritize the objectives set in the officially approved breeding programs for each breed.

The physiological evaluations were carried out during the finals of the Dressage YHST events, held at the equestrian center “Dos Lunas Polo Dressage” in San Martín del Tesorillo, in Cádiz (Spain) in the following dates: on 14–15 September in both 2022 (with 69 PRE horses participating) and 2023 (84 PRE horses participating), and 18–20 September 2024 (120 PRE horses).

Horses usually arrived one day before the competition and often remained for at least a few hours or 1 day after the events ended. They were housed in 3 × 3 m boxes, with fresh hay and concentrate twice a day and water *ad libitum*. During the tests, horses were required to perform specific Dressage movements under the rider, varying the degrees of difficulty according to their age. All horses were subjected to the same rest and exercise regime prior to the performance test.

### 2.3 Facial hair whorls (FHW)

Hair whorls were analyzed in two ways:

- Based on their location on the head:

- FHW above the eye line (FHW_above).
- FHW on the eye line (FHW_middle).
- FHW below the eye line (FHW_below).

- Based on the number of hair whorls on each horse:

- No FHW present.
- A single FHW.
- More than one FHW.

**Figure 1:**
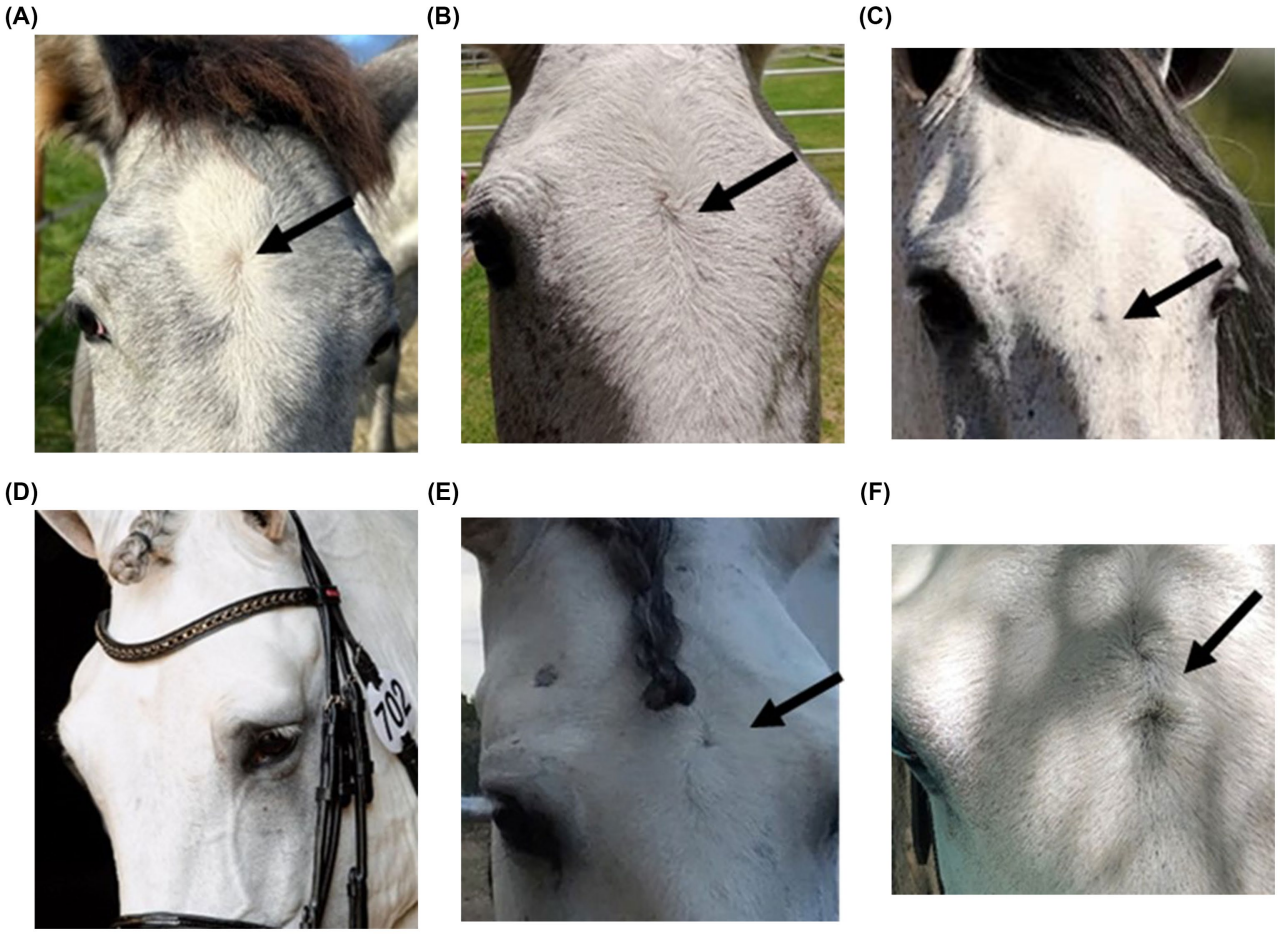
Examples of locations and number of facial hair whorls (FHW) located on the head of different Pura Raza Española horses. Based on their location on the head **(A)** FHW above the eye line (FHW above), **(B)** FHW on the eye line (FHW_middle), **(C)** FHW below the eye line (FHW_below). Based on the number of hair whorls on each horse **(D)** no FHW present, **(E)** a single FHW, **(F)** more than one FHW.

Some photos of PRE horses with different numbers and locations of hair whorls located on the head were presented in Figure 1. FHW assessment was performed by direct visual inspection of the head under adequate lighting to minimize the risk of misclassification.

### 2.4 Infrared thermography (IRT)

Ocular temperature images with IRT were always taken by the same person using a portable infrared thermographic camera (FLIR E90. FLIR Systems AB, Danderyd, Sweden). Photographs of the left eye of each animal were taken from a 90° angle and 1-meter distance. Several images were taken per animal and collection period. Later, the image that provided the most optimal operating conditions for analysis was selected. The emissivity of the camera was set to 0.98.

Besides, ocular temperature (°C) was assessed using FLIR Tools 6.0.17046.1002 software (FLIR Systems AB, Danderyd, Sweden). With this software, the maximum temperature was obtained within an oval-shaped area drawn around the eye’s caruncle, including approximately 1 cm around it. To calibrate the camera images analyzed with the software, environmental temperature and relative humidity were recorded using a digital thermo-hygrometer (Extech 44,550, Extech Instruments, Nashua, New Hampshire) each time an ocular temperature sample was taken. All thermographic images were acquired under standardized field conditions, avoiding direct solar radiation on the eye region and extreme lighting conditions, and following established recommendations for infrared thermography assessment in outdoor environments.

Each horse was photographed at three specific moments during the days of each YHST final, in order to reflect different physiological states:

- PRE_IRT: assessed 2–3 h before the test, with the horse at rest in its box. It showed the stress resting baseline of the horse.
- DUR_IRT: assessed immediately after finishing the test, at the arena. It showed the acute post-exercise stress response of the horse.
- POST_IRT: assessed approximately 2–3 h after the test, with the horse again resting in its box. It showed the post-exercise stress of the horse.

From these data, PETI (DUR_IRT – PRE_IRT) and LETI (DUR_IRT – POST_IRT) were calculated. Where PETI assessed the effort stress previous to the competition and LETI the recovery stress from the competition. The best-quality images were then selected for this analysis.

### 2.5 Behavioral data

Behavioral assessment was based on a survey conducted by the rider evaluating seven general behavioral traits in the context of the equestrian competitions, on a scale from 1 to 5, with 1 = minimal intensity, 2 = below average, 3 = average, 4 = above average and 5 = maximum intensity. Behavioral data were collected during the competition period, generally after the horse had completed its test. However, respondents were asked to rate the horse’s general behavioral characteristics based on their regular training experience, rather than its behavior during that single competitive performance. Each horse was evaluated by its regular rider or primary caretaker, who was familiar with the horse’s behavior through daily training and management; in some cases, the same person evaluated more than one horse.

The seven behavioral traits assessed were adapted from previous validated surveys (1, 25):

- *Aggressiveness*: Challenging or sometimes violent behavior towards people or other horses, either direct (e.g., bites or kicks) or indirect (threatening gestures).
- *Dominance*: Constant display of leadership over other horses or people (not always aggressive).
- *Nervousness*: General excitability not tied to a specific stimulus or novel circumstance.
- *Excitability*: Reaction to novel situations or objects.
- *Learning Ability*: Horse’s ability to acquire, retain, and reproduce new behaviors or exercises taught by the person in charge.
- *Cooperation:* The horse’s attitude towards working with the rider during training or competition.
- *Endurance*: Referred to as stamina under pressure. Although not a behavioral trait in the strict sense, endurance was included to reflect both physical stamina and behavioral aspects such as stress coping and persistence.

### 2.6 Statistical analyses

All statistical procedures were performed using R software (version 4.3.2; R Core Team, 2025).

#### 2.6.1 Preliminary analyses

The Kolmogorov–Smirnov test (*ks.test*) was used to assess the normality of thermographic variables. Descriptive statistics, including absolute and relative frequencies of facial hair whorls (FHW), were computed using the table and *prop.table* functions.

#### 2.6.2 Modelling associations between FHW, behavior, and thermographic traits

A series of linear models were fitted to explore the relationships among morphological, behavioral, and physiological traits. First, the effect of the number of FHW in three anatomical regions (above, on, and below the eye line) on each thermographic variable was assessed. These models were fitted using the *lm function*. When significant differences were found, Duncan’s multiple range test was applied for *post hoc* comparisons, using the *duncan.test function* (*agricolae package*).

Second, models were constructed to test the effect of behavioral traits (main effects and selected interactions) on thermographic responses. Predictions were visualized using *ggplot2* and *interact_plot* (*interactions package*).

Third, generalized linear models with a Gaussian distribution (*glm, family = gaussian*) were used to assess whether FHW counts predicted behavioral trait scores.

#### 2.6.3 Cluster analysis

A k-means cluster analysis was performed to identify profiles based on standardized thermographic, behavioral, and FHW variables. Prior to clustering, all variables were standardized (mean = 0, SD = 1) to ensure comparability. The optimal number of clusters (*k* = 3) was determined using the elbow method (*fviz_nbclust*, *factoextra package*). Clustering was conducted with the *kmeans function*, and the resulting groups were interpreted in terms of reactivity, FHW distribution, and stress response.

#### 2.6.4 Principal component analysis (PCA)

To explore multivariate relationships and reduce dimensionality, a Principal Component Analysis was conducted on standardized data using the *prcomp* function. The first five principal components explained 70.5% of the variance, and the first three (49.2%) were retained for interpretation. Variable loadings were extracted using the *summary* and *loadings* functions from the *psych package*, and individuals were visualized in the reduced space using *fviz_pca_ind*, with clusters overlaid via *fviz_cluster* (*factoextra*).

## 3 Results

Table 1 shows the distribution of facial hair whorls (FHW) across different regions of the head in 98 PRE horses. The highest frequency of FHW was observed along the eye line (60.2%), followed by FHW located below the eye line (37.7%), and while the least frequent were those positioned above the eye line (18.4%). A total of 39.8% of the horses did not present any visible FHW, and no individuals exhibited more than one FHW below the eye line. Percentages referred to horses within each FHW category.

**Table 1:** Number and location of facial hair whorls in the Pura Raza Española horses analyzed.

Hair whorl number	Hair whorl location		
	**Above eye line**	**On eye line**	**Below eye line**
No hair whorl	80 horses (81.6%)	39 horses (39.8%)	61 horses (62.2%)
One hair whorl	15 horses (15.3%)	54 horses (55.1%)	37 horses (37.8%)
More than one hair whorl	3 horses (3.1%)	5 horses (5.1%)	0 horses (0%)

The distribution of behavioral traits in the analyzed population is presented in Table 2. In general, horses exhibited low levels of *aggressiveness* (Class 1: 57.1%), *dominance* (Class 1: 41.8%), and *nervousness* (Class 1: 33.7%). *Excitability* was mostly mild (Class 2: 36.7%). In contrast, high values were reported for *learning ability* (Classes 4 and 5: 78.5%), *cooperation* (Classes 4 and 5: 78.5%), and *endurance* (Class 5: 55.1%). Prior to conducting the statistical analyses involving IRT variables, normality was assessed using the Kolmogorov–Smirnov test for PRE_IRT, DUR_IRT, POST_IRT, PETI and LETI variables, (results not shown). All IRT variables showed *p*-values > 0.05, confirming a normal distribution.

**Table 2:** Frequency distribution of behavioral traits in the Pura Raza Española horses analyzed.

Behavioral Variables	Class 1	Class 2	Class 3	Class 4	Class 5
Aggressiveness	56 (57.1%)	17 (17.4%)	14 (14.3%)	7 (7.1%)	4 (4.1%)
Dominance	41 (41.8%)	24 (24.5%)	19 (19.4%)	8 (8.2%)	6 (6.1%)
Nervousness	33 (33.7%)	29 (29.6%)	20 (20.4%)	14 (14.3%)	2 (2.0%)
Excitability	28 (28.6%)	36 (36.7%)	15 (15.3%)	17 (17.4%)	2 (2.0%)
Learning ability	3 (3.1%)	2 (2.0%)	16 (16.3%)	36 (36.7%)	41 (41.8%)
Cooperation	1 (1.0%)	8 (8.2%)	12 (12.2%)	31 (31.6%)	46 (46.9%)
Endurance	2 (2.0%)	3 (3.1%)	12 (12.2%)	27 (27.6%)	54 (55.1%)

Behavioral traits were scored on a 5-point scale, where Class 1 = minimal intensity, Class 2 = below average, Class 3 = average, Class 4 = above average and Class 5 = maximum intensity.

Table 3 presents the descriptive statistics for the thermographic variables. It should be noted that ocular temperature was not measured as repeated technical replicates, but at three physiologically distinct moments reflecting different states: resting baseline (PRE_IRT), acute post-exercise response (DUR_IRT), and post-exercise recovery (POST_IRT). Mean temperature at rest (PRE_IRT) was 35.3 °C. After completing the test (DUR_IRT), temperature increased to 36.4 °C. Two hours post-test (POST_IRT), temperature slightly decreased to 36.2 °C. Standard deviations ranged from 0.68 (POST_IRT) to 1.18 (PETI).

**Table 3:** Descriptive statistics for the thermographic variables.

Analyses	PRE_IRT	DUR_IRT	POST_IRT	PETI	LETI
Mean ± s.e	35.26 ± 0.087	36.37 ± 0.113	36.15 ± 0.069	1.10 ± 0.119	0.21 ± 0.108
s.d.	0.861	1.123	0.682	1.181	1.073

Where PRE_IRT = Ocular temperature assessed approximately 2–3 h before the test, with the horse at rest in its box; DUR_IRT = Ocular temperature assessed immediately after finishing the test, at the arena; POST_IRT = Ocular temperature assessed approximately 2–3 h after the test, with the horse again resting in its box; PETI = Calculated as (DUR_IRT – PRE_IRT); LETI = Calculated as (DUR_IRT – POST_IRT); s.e. = standard error; s.d. = standard deviation.

A General Linear Model (GLM) was applied to assess the influence of FHW number and location on IRT variables (Table 4). When statistical significance was detected, Duncan’s *post hoc* test was used for mean comparisons. Significant differences were observed for FHW located on the eye line in DUR_IRT, PETI and LETI variables. Specifically, horses with more than one FHW on the eye line showed significantly higher IRT temperatures (DUR_IRT = 37.7 °C), greater ocular temperature increases before and after competition (PETI = 2.8 °C and LETI = 1.6 °C, respectively) than horses with none (DUR_IRT = 36.2 °C; PETI = 1.0 °C; LETI = 0.2 °C) or a single FHW (DUR_IRT = 36.34 °C PETI = 1.0 °C; LETI = 0.1 °C) in this position.

**Table 4:** General linear model analysis of the effect of FHW number/location on thermographic variables.

Region	Score	PRE_IRT	DUR_IRT	POST_IRT	PETI	LETI
Above the eye line	0	35.21 ± 0.094 (0.842)	36.31 ± 0.123 (1.103)	36.15 ± 0.077 (0.689)	1.10 ± 0.136 (1.214)	0.16 ± 0.119 (1.060)
	1	35.53 ± 0.264 (1.021)	36.49 ± 0.332 (1.288)	36.17 ± 0.182 (0.704)	0.96 ± 0.280 (1.083)	0.32 ± 0.299 (1.158)
	>1	35.29 ± 0.072 (0.124)	37.19 ± 0.313 (0.543)	35.96 ± 0.295 (0.510)	1.89 ± 0.246 (0.426)	1.22 ± 0.288 (0.499)
	*p*-value	0.4388	0.3775	0.8881	0.4649	0.2229
On the eye line	0	35.20 ± 0.159 (0.993)	**36.23 ± 0.166** ^**a**^ **(1.038)**	35.99 ± 0.120 (0.752)	**1.03 ± 0.171**^**a**^ **(1.069)**	**0.24 ± 0.153**^**a**^ **(0.955)**
	1	35.34 ± 0.100 (0.736)	**36.34 ± 0.155**^**a**^ **(1.136)**	36.27 ± 0.084 (0.614)	**1.00 ± 0.160**^**a**^ **(1.173)**	**0.07 ± 0.149**^**a**^ **(1.099)**
	>1	34.95 ± 0.487 (1.088)	**37.71 ± 0.406**^**b**^ **(0.909)**	36.12 ± 0.307 (0.686)	**2.76 ± 0.470** ^**b**^ **(1.052)**	**1.60 ± 0.340**^**b**^ **(0.760)**
	*p*-value	0.5368	**0.0189**	0.1674	**0.0045**	**0.0085**
Below the eye line	0	35.32 ± 0.107 (0.839)	36.38 ± 0.148 (1.157)	36.23 ± 0.077 (0.603)	1.06 ± 0.158 (1.236)	0.15 ± 0.143 (1.115)
	1	35.16 ± 0.148 (0.899)	36.34 ± 0.178 (1.081)	36.02 ± 0.129 (0.785)	1.18 ± 0.181 (1.098)	0.32 ± 0.165 (1.005)
	*p*-value	0.3785	0.8682	0.1331	0.6292	0.4376

Mean ± s.e. (s.d.) are shown. Duncan’s *post hoc* mean comparisons were applied. Where PRE_IRT = Ocular temperature assessed approximately 2–3 h before the test, with the horse at rest in its box; DUR_IRT = Ocular temperature assessed immediately after finishing the test, at the arena; POST_IRT = Ocular temperature assessed approximately 2–3 h after the test, with the horse again resting in its box; PETI = Calculated as (DUR_IRT – PRE_IRT); LETI = Calculated as (DUR_IRT – POST_IRT); s.e. = standard error; s.d. = standard deviation. Different superscript letters indicate statistically significant differences (*p* < 0.05) between groups. Bolded values indicate statistically significant results (*p* < 0.05).

To assess the relationships between behavioral traits (individually and pairwise interactions) and thermographic variables, a GLM was implemented using behavioral variables as predictors and thermographic measures as dependent variables (results not shown). Behavioral variables were considered as stable trait-like characteristics of the horses, whereas thermographic variables reflected acute physiological responses to the competition. Accordingly, the analyses were designed to assess associations between behavioral profiles and thermographic responses, rather than direct temporal causality. When behavioral variables were analyzed individually, only *endurance* had a statistically significant effect on the PETI (*p* < 0.05). In contrast, when examining interactions between pairs of behavioral variables, the interaction between *learning ability* and *endurance* showed a statistically significant influence on the thermographic variables DUR_IRT (*p* < 0.01), PETI (*p* < 0.01), and LETI (*p* < 0.01). The interaction between *aggressiveness* and *nervousness* showed statistically significant results for POST_IRT (*p* < 0.05) and LETI (*p* < 0.01). Additionally, the interaction between *learning ability* and *cooperation* showed a statistically significant effect only for LETI (*p* < 0.05).

**Figure 2:**
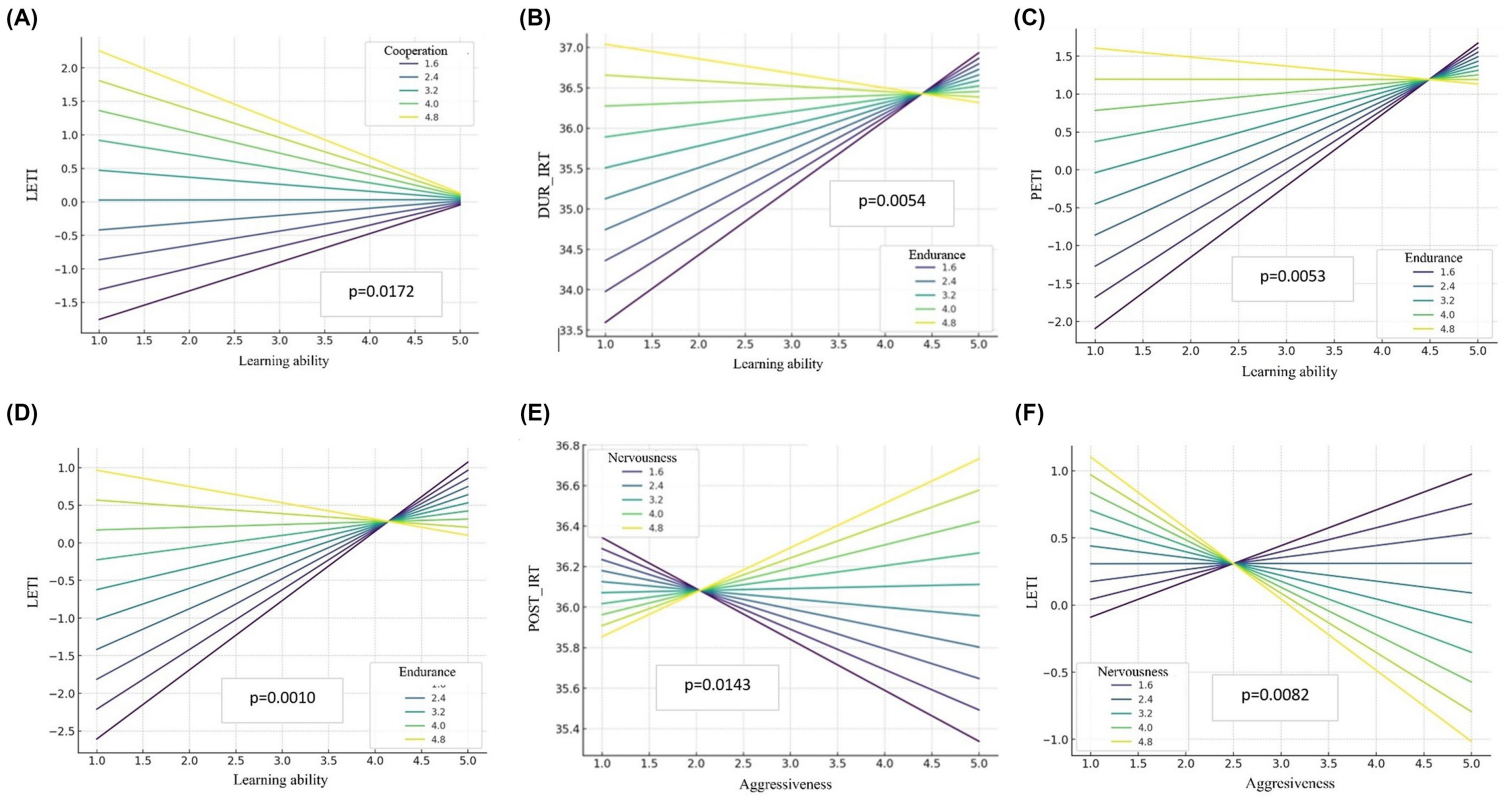
General linear model (GLM) interaction plots showing the combined effects of behavioral traits on ocular thermographic variables. **(A)** Effect of learning ability × cooperation interaction on LETI; **(B)** effect of learning ability × endurance interaction on DUR_IRT; **(C)** effect of learning ability × endurance interaction on PETI; **(D)** effect of learning ability × endurance interaction on LETI; **(E)** effect of aggressiveness x nervousness interaction on POST_IRT; **(F)** effect of aggressiveness × nervousness interaction on LETI, where DUR_IRT = ocular temperature assessed immediately after finishing the test, at the arena; POST_IRT = ocular temperature assessed approximately 2–3 h after the test, with the horse again resting in its box; PETI = calculated as (DUR_IRT – PRE_IRT); LETI = calculated as (DUR_IRT – POST_IRT).

For those behavioral variable interactions that showed significant effects on thermographic outcomes (*learning ability* x *endurance*, *aggressiveness* x *nervousness* and *learning ability* x *cooperation*), a more detailed analysis was conducted in Figure 2. Predicted values based on the adjusted models were generated and interaction plots were constructed to visualize how levels of behavioral traits jointly influenced thermographic variables, considering their combined effects. These plots are intended to provide a qualitative visualization of interaction trends rather than a precise quantitative comparison of predicted values.

Regarding the effect of the interaction between *learning ability* and *cooperation* on LETI (Figure 2A), results showed that, at high levels of *cooperation*, LETI values tended to slightly decrease as learning increased, indicating a more efficient thermal recovery. At low levels of *cooperation*, the effect of learning was flatter or even reversed, suggesting that these horses exhibited slower recovery regardless of their learning ability. For the interaction between *learning* and *endurance* on DUR_IRT (Figure 2B), the graph showed that, as *learning ability* increased, DUR_IRT also tended to rise, particularly at high levels of *endurance*. In contrast, at low *endurance* levels, the effect of *learning ability* was more stable, indicating reduced sensitivity in these combinations.

When the interaction between *learning ability* and *endurance* on PETI was assessed (Figure 2C), PETI values tended to be higher with elevated levels of both traits. In contrast, horses with low *endurance* exhibited a lower increase in PETI, even if their *learning ability* was high. The interaction between *learning* ability and *endurance* on LETI (Figure 2D) revealed an inverse trend compared to the other variables: as *learning ability* and *endurance* increased, LETI values decreased, indicating faster thermal recovery. At low *endurance* levels, this trend was less pronounced.

Regarding the interaction between *aggressiveness* and *nervousnes*s on POST_IRT (Figure 2E) results showed that, as *aggressiveness* increased, POST_IRT values varied depending on the level of *nervousness*. High *nervousness* amplified the effect of a*ggressiveness*, resulting on higher POST_IRT values. Conversely, at low levels of *nervousness*, the impact of *aggressiveness* was attenuated. Finally, the interaction between *aggressiveness* and *nervousness* on LETI (Figure 2F) showed that, as LETI values decreased as both *aggressiveness* and *nervousness* increased. High levels of both traits were associated with lower LETI values, indicating slower recovery. However, the effect of *aggressiveness* on LETI was less apparent in horses with low *nervousness*.

To assess the effect of the number of FHW in different locations on behavioral variables, General Linear Models (GLM) were used (results not shown). Each behavioral variable was modeled as a dependent variable, with the number of FHW (categorized into three classes by location) used as a predictor. *Statistically* significant associations were identified in PRE horses between *aggressiveness* and FHW located along the eye line (*p* < 0.05). Additionally, *nervousness* showed a moderate association (*p* < 0.01) whereas other traits (*excitability*, *learning ability, cooperation* and *endurance*) showed strong associations (*p* < 0.001) consistent across FHW locations.

To identify patterns among thermographic, behavioral, and FHW variables, a cluster analysis was performed. Variables were standardized before clustering; however, Table 5 presents raw mean values (back transformed to the original scale) to facilitate biological interpretation. Each cluster was characterized based on the means values of the variables obtained through k-means clustering, allowing for the interpretation of intergroup differences. Cluster 0 predominantly included horses with medium to high levels of *nervousness* and *excitability*. These horses also showed elevated ocular temperatures at the end of the Dressage test (DUR_IRT), along with higher thermal increments both before and after exercise (PETI and LETI, respectively). Additionally, they presented a greater number of FHW located above and on the eye line. Cluster 1 grouped horses with high levels of *cooperation, learning ability* and *endurance.* These individuals showed lower PETI values, suggesting reduced stress-related thermal responses, and lower LETI values, indicating faster thermal recovery. This cluster also included the higher number of FHW located below the eye line. Finally, Cluster 2 was characterized by horses with high levels of *aggressiveness, dominance, nervousness* and *excitability*, yet surprisingly, they exhibited lower overall thermographic responses in both PETI and LETI compared to the other clusters. The dissociation observed in Cluster 2 between high behavioral reactivity (aggressiveness, dominance, nervousness) and comparatively lower thermographic responses may reflect different coping styles, in which overt behavioral expression is not necessarily accompanied by heightened physiological stress activation. This profile may suggest a dissociation between behavioral reactivity and physiological stress expression in some individuals.

**Table 5:** Standardized mean values of thermographic, behavioral, and facial hair whorl variables across the three clusters identified by *k*-means clustering.

proVariables	Clusters		
	Cluster 0	Cluster 1	Cluster 2
PRE_IRT	35.31	35.18	35.37
DUR_IRT	**37.36**	36.16	35.59
POST_IRT	36.28	36.31	35.69
PETI	**2.06**	0.98	0.22
LETI	**1.09**	−0.15	−0.10
Aggressiveness	1.61	1.26	**3.21**
Dominance	1.71	1.74	**3.33**
Nervousness	2.61	1.54	**3.04**
Excitability	2.75	1.56	**3.00**
Learning	3.71	**4.65**	3.58
Cooperation	3.89	**4.69**	3.42
Endurance	4.07	**4.56**	4.08
FHW_AboveEyes	**1.43**	1.09	1.21
FHW_OnEyeLine	**1.89**	1.54	1.58
FHW_BelowEyes	1.21	**1.48**	1.38

Where PRE_IRT = Ocular temperature assessed approximately 2–3 h before the test, with the horse at rest in its box; DUR_IRT = Ocular temperature assessed immediately after finishing the test, at the arena; POST_IRT = Ocular temperature assessed approximately 2–3 h after the test, with the horse again resting in its box; PETI = Calculated as (DUR_IRT – PRE_IRT); LETI = Calculated as (DUR_IRT – POST_IRT). FHW = facial hair whorls. Numbers in bold indicate the cluster assigned for that variable. Numbers in bold indicate the cluster assigned for that variable. Although clustering was performed on standardized data, raw mean values (original scale) are presented here to facilitate interpretation.

**Figure 3:**
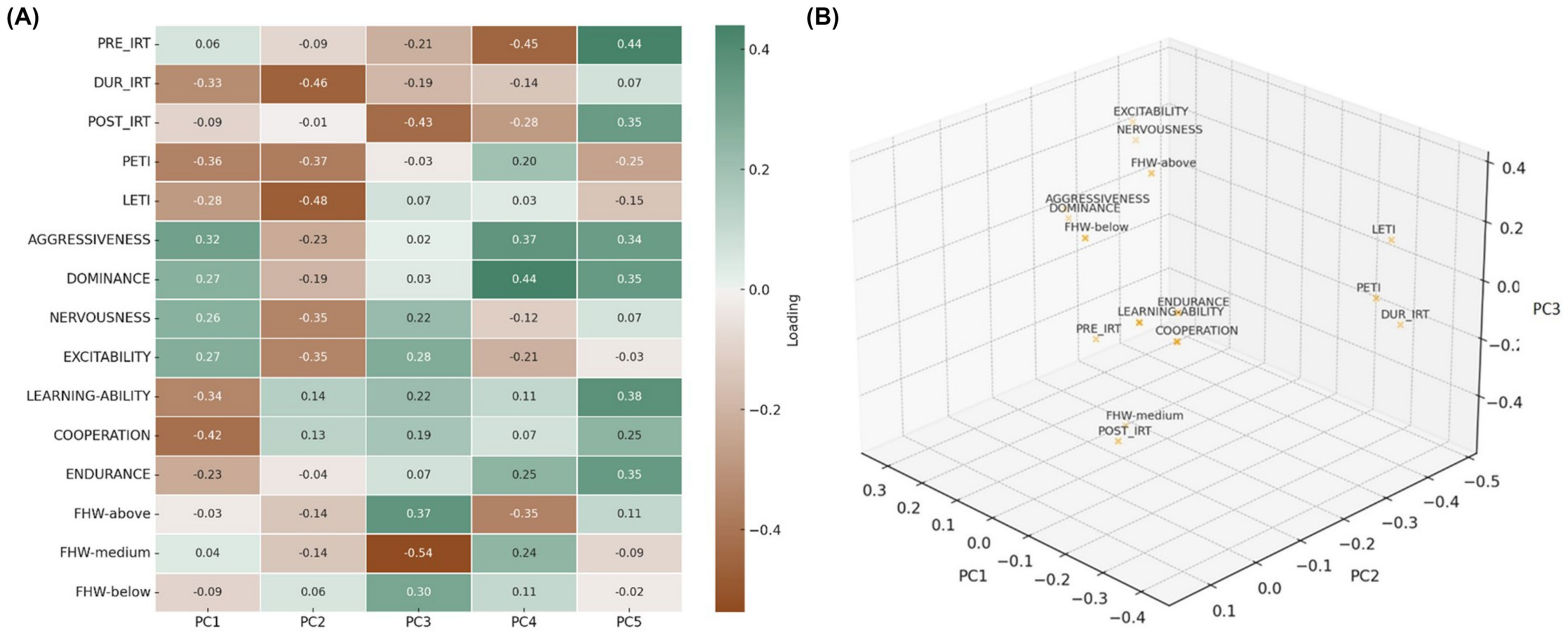
Principal component analysis (PCA) applied to thermographic, behavioral, and facial hair whorl variables. **(A)** Variable loadings on the first five principal components (PC1–PC5), represented as a heatmap. **(B)** Three-dimensional projection of individuals on the PCA space defined by PC1, PC2, and PC3, where PRE_IRT = ocular temperature assessed approximately 2–3 h before the test; DUR_IRT = ocular temperature assessed immediately after finishing the test; POST_IRT = ocular temperature assessed approximately 2–3 h after the test; PETI = calculated as (DUR_IRT – PRE_IRT); LETI = calculated as (DUR_IRT – POST_IRT); FHW below = facial hair whorls located below the eyeline; FHW medium = facial hair whorls located on the eyeline; FHW above = facial hair whorls located above the eyeline.

Figure 3 showed the loadings of the different analyzed variables (FHW, thermographic and behavioral variables) on the various principal components and presented a graphical representation of the first three components, with the variables grouped due to these components (PC1, PC2 and PC3). PC1 (19.6%), PC2 (17.1%) and PC3 (12.5%), together accounted for over 49% of the total variance. Up to PC5, approximately 70.5% of the variance was explained. In Figure 3A, PC1 contrasted *dominance/aggressiveness* (positive loadings) with *cooperation*, *learning ability*, and higher thermographic reactivity (negative loadings; PETI and LETI), capturing a dominance–docility/learning gradient. Besides, PC2 showed negative loadings on thermographic variables (DUR_IRT, LETI, PETI) and reactive behaviors (*nervousness* and *excitability*), reflecting physiological and emotional reactivity. Furthermore, PC3 indicated that FHW located in the middle region (eye line) had strong negative loadings, while those at the top of the head had positive ones. Therefore, PC3 would capture patterns in FHW distribution. The thermographic variable POST_IRT also loaded on this component, potentially reflecting a post-exercise thermal response. PC4 and PC5 were more difficult to interpret but appeared to represent combinations of behavioral traits (*dominance* and *endurance*) and thermographic measures. Notably, the FHW-medium showed its highest loading on PC4, suggesting that this axis may better capture the potential influence of this specific FHW location on thermographic and behavioral responses. This reinforces its relevance as a morphological marker of interest.

On the other hand, the 3D plot in Figure 3B showed that, as regards behavioral variables, *aggressiveness*, *dominance*, *nervousness*, and *excitability* were very close in the 3D space, indicating that they were related to each other. These variables defined a profile of greater emotional reactivity or impulsiveness. Besides, *cooperation* and *learning ability* were clustered on the opposite side of PC1 and in the positive zone of PC2 and PC3, suggesting a profile of collaboration, learning, and less conflict. Thus, PC1 appears to separate more reactive and dominant animals (positive values) from more collaborative and adaptive animals (negative values). As regards physiological variables (IRT), DUR_IRT and PETI were in the same area of PC1 and PC2, showing the increase in temperature during exercise. On the other hand, POST_IRT and LETI appeared further apart, indicating thermal recovery or maintenance after the test. Besides, *endurance* appeared close to these variables, possibly indicating physiological resistance to exercise. Thus, PC2 could be capturing the intensity of the physiological response to exercise, and PC3, the recovery ability. Finally, the FHW on eye line (FHW medium) appeared clearly separated in PC3, with a very negative value, whereas the FHW below eyes (FHW below) showed a very positive value and the FHW above eyes (FHW above) was in an intermediate position in this same component (PC3). Hence, PC3 appears to capture a vertical gradient in the position of the FHW, suggesting that this feature could be related to thermal or behavioral profiles. To sum up, PC1 seemed to represent a behavioral profile, differentiating between *reactivity* and *dominance* versus *cooperation* and *learning ability*. PC2 component showed the intensity of the physiological response to stress, whereas PC3 showed the differences in thermal recovery and FHW position.

## 4 Discussion

Facial hair whorls (FHW) in horses have long been considered congenital markers with potential behavioral associations in horses (26, 27). In the present study, their distribution was analyzed in a homogeneous sample of 98 Pura Raza Española horse (PRE), all gray-coated, aged between 4 and 6 years old, and evaluated within the context of functional selection for Dressage. This specific context differs considerably from previous large-scale surveys such as that of Encina et al. (19), which examined over 43,398 PRE horses of various coat colors, ages, and functional orientations. Besides, the pattern described in Table 1 of this study also diverged substantially from that reported by Encina et al. (19), who found a much higher prevalence of FHW located below the eye line, both in the general population and in gray-coated horses. While our sample showed a predominance of FHW located at eye level, Encina et al. (19) reported the lower regions of the head as the most frequent, with percentages exceeding 49% in the lower right area and over 47% in the lower left. This discrepancy may be attributed to the specific characteristics of the population studied. The uniformity in coat color (gray), the limited age range, and the selection for functional suitability in Dressage introduce potential sources of bias that must be considered when interpreting the results.

Our data also indicated a remarkably high proportion of individuals (39.8%) without any visible FHW, which is atypical when compared to previous studies in horses [e.g., Arabian horses by Abdel-Azeem and Emeash (28), PRE horses by Encina et al., (19) or even in other domestic species such as cattle (12)]. Although coat characteristics or grooming could potentially influence the ease of visual detection of facial hair whorls, FHW assessment in this study was based on direct inspection under standardized conditions, and no evidence suggested systematic misclassification due to these factors. This suggests that the distribution pattern observed, both in terms of number and location, may not reflect that of the general PRE population. It is plausible that horses selected for Dressage, which favors specific behavioral and functional traits, exhibit a different expression of morphological markers such as FHW. Rather than selection pressures directly altering the phenotypic expression of hair whorls, it is more likely that the animals chosen for their desirable performance profiles tend to share certain morphological traits consequently.

With respect to behavioral traits, Table 2 showed a generally homogeneous profile, consistent with the traditional image of the PRE as a balanced, docile, and functional horse, suitable for disciplines such as Dressage (8). Regarding the methodology used to measure behavioral traits in the PRE horses, there is a major drawback concerning behavioral variables—they are very subjective when it comes to measurement. The evaluation of behavior was conducted through a questionnaire using a 5-point scale, which—despite its inherent subjectivity—has been validated as a useful tool in large-scale population studies (29, 30). While individual perception may vary, the systematic application of this methodology helped identify consistent behavioral patterns across the sample.

Regarding the physiological profile of the population, Table 3 presented the descriptive statistics of ocular temperature measured at different time points during the Dressage competition (before, during, and after the test), using infrared thermography (IRT). These values fell within the normal physiological range for adult horses, confirming that the animals did not show hyperthermia or extreme responses. These results aligned with those described by Valera et al. (3) and Bartolomé et al. (31) in Spanish Sport Horses (CDE), who observed similar ocular temperature increases after Show Jumping competitions. They were also consistent with data from Sánchez et al. (8) in PRE horses competing in Dressage test. Thus, previous studies supported the use of ocular temperature assessed with infrared thermography as a sensitive and non-invasive tool for characterizing physiological reactivity in PRE horses.

This study explored, in an integrated manner, the relationship between FHW (location and number), behavior (assessed with questionnaires), and the physiological stress response (measured with ocular temperature assessed by infrared thermography) in young PRE horses selected for Dressage. Using GLM, PCA, and cluster analyses, functional profiles were identified that might be relevant for horse selection and management.

One of the most significant findings was reflected in Table 4, where it was observed that the number of FHW along the eye line significantly influenced ocular temperature just after the Dressage test (DUR_IRT) and both PETI and LETI increases. Specifically, horses with more than one FHW in this location showed higher temperature values in all three variables. These statistically significant (*p* < 0.05) and physiologically relevant differences suggested that a higher number of FHW in this region might be associated with greater physiological stress reactivity. This heightened thermal response could indicate stronger sympathetic nervous system activation, reflecting acute alertness or stress (3, 31), and aligns with previous findings linking multiple FHW to more reactive or sensitive temperaments (17). This result was particularly noteworthy because it is not the high-positioned FHW, as previously proposed by Grandin et al. (12) and Grandin and Deesing (17), but rather those at eye level that are linked to greater reactivity, possibly due to visual interference with stress stimuli or the sensory sensitivity of that region.

A crucial interaction for understanding thermal stress response was highlighted in the first GLM analysis, which evaluated the effect of behavioral variables on thermographic responses. A combination of high *aggressiveness* and *nervousness* seemed to negatively affect post-stress recovery, while *learning ability* and *endurance* influenced how temperature was regulated during and after the test. These findings supported the idea that equine personality affects physiological stress responses, as Sauer et al. (9) already had. The second GLM model (results not shown) focused on the relationship between FHW characteristics and behavioral traits. Horses with FHW above the eyes showed higher scores in *nervousness*, whereas those with FHW at eye level showed a stronger *aggressiveness* response. These consistent trends suggested that FHW’s location could be related to reactive personality traits. This aligns with findings by Górecka et al. (27), who reported that horses with higher FHW positions were less manageable.

Besides, Figure 2 illustrated the effects of interactions between behavioral trait ranges on thermal responses in PRE horses. The results showed that not only individual traits but also specific combinations significantly influence physiological stress modulation. Specifically, PRE horses with high *endurance* and *learning ability* experienced greater temperature increases just after the test (DUR_IRT), possibly due to a more active participation or higher physical demands. This behavioral combination was also associated with higher PETI values, suggesting greater general thermal activation linked to both effort and emotional stress from the test. However, these same individuals showed lower LETI values, possibly indicating more efficient post-test thermal recovery, likely due to better physical condition or stress management.

Similarly, the interaction between learning ability and cooperation was related to lower LETI, suggesting this combination fosters better emotional regulation and faster physiological recovery. These horses seem better adapted to demanding situations, both physically and emotionally. In contrast, the interaction between aggressiveness and nervousness showed the opposite trend, with higher POST_IRT values, possibly reflecting difficulty in recovery or lingering stress after the test. LETI values also tended to be higher in these horses, reinforcing the hypothesis that reactive temperaments hinder thermal recovery after exercise and may impair stress management in competitions.

These findings are consistent with earlier studies, which noted that emotionally reactive animals tend to struggle with stress regulation, whereas more cooperative and trainable horses recover more efficiently from stressful situations (17). Taken together, these results support a conceptual framework in which facial hair whorl patterns act as stable morphological markers associated with behavioral profiles, which in turn are linked to differential physiological stress responses. Rather than implying a direct causal or neurobiological connection, the observed associations suggest that FHW may reflect early developmental factors that co-occur with temperament traits influencing autonomic reactivity during challenging situations. In this context, ocular temperature assessed by infrared thermography provides an objective physiological output that complements behavioral assessment, allowing a more integrative interpretation of stress responsiveness in sport horses. Similar associations between stable behavioral traits and physiological stress responses have been reported in other horse populations and breeds, supporting the broader relevance of integrative approaches combining temperament assessment and objective physiological indicators (32–34). Similarly to Visser et al. (25), the results support the hypothesis that behavioral profiles marked by high nervousness or aggressiveness complicate post-stress physiological recovery in equines, helping to define distinct equine personality types. Altogether, these results suggested that simultaneously evaluating behavioral traits such as cooperation, learning, and nervousness could be very useful in optimizing selection and training programs for sport horses.

After analyzing specific interactions between behavioral traits and ocular temperature measured via infrared thermography, the k-means analysis grouped PRE horses into three clusters integrating all analyzed variables (Table 5): FHW, behavior, and temperature, with the aim of identifying shared patterns among the evaluated horses. Cluster 0 results aligned with Grandin and Deesing (17), who indicated that hair whorls located higher on the head or in double form were associated with more nervous or reactive temperaments. It also matches findings from Bartolomé and Cockram (35), who noted that reactive temperaments and low physiological resilience may compromise sports performance under stress. Cluster 1, in turn, supported Bartolomé and Cockram’s (35) hypothesis that balanced, resilient temperaments enhance physiological and emotional adaptability in competition. Besides, horses in this cluster generally had FHW below or at eye level. Similarly, Grandin and Deesing (17) noted that lower-head whorls correlated with greater docility and ease of handling. Last, Cluster 2 results suggested that *dominance* and *aggressiveness* might not be as directly influenced by FHW position or number as other behavioral traits. According to Visser et al. (25), dominant-related temperament may be physiologically independent from stress reactivity, which could explain the weaker morphological association between FHW patterns, and *dominant* or *aggressive* behaviors observed in this group. This pattern supports previous evidence suggesting that dominant or aggressive temperaments may operate through behavioral strategies that are partially independent from autonomic stress responses, leading to a weaker correspondence between external behavior and physiological indicators of stress.

Finally, these patterns were supported by the principal component analysis from Figure 3. The first component (PC1) reflected a gradient between *cooperation* and *learning ability* versus *aggressiveness* and *dominance*, while the second (PC2) represented physiological and emotional reactivity tied to thermal and behavioral variables like *nervousness* and *excitability*. PC3, in turn, captured the distribution of FHW across vertical facial zones, separating those located above, at, or below the eye line. This three-dimensional graph allowed us to visualize how the behavioral, physiological, and FHW variables clustered together.

The observed associations suggested, first, that FHW could be related to the type of thermal or behavioral response. This observation is consistent with existing evidence suggesting a morphological basis for temperament. Grandin et al. (12) found that the location of hair whorls on the face of cattle was significantly associated with differences in temperament; animals with higher hair whorls tended to be more excitable. Likewise, in horses, Górecka et al. (27) demonstrated correlations between FHW positions and behavioral expressions, indicative of temperament types.

Second, different profiles of *reactivity* vs. *cooperation*, and intense thermal response vs. recovery were found. These results align with multidimensional models of animal temperament. As described previously, Górecka et al. (27) reported that horses’ hair whorl positions were significantly related to behavioral reactivity during handling, and that these differences could inform management and handling practices. Furthermore, Koolhaas et al. (36) emphasized the importance of recognizing both physiological and behavioral axes in evaluating coping styles and stress resilience in animals.

Third and last, the location of FHW on the head appeared to have an important influence on the PC3 dimension, which distinguished between high, medium, and low hair whorl positions, opening the door to future interpretations of its value as an external marker of temperament or physiological sensitivity. Notably, the strongest loading for FHW-medium was observed in PC4, a component not visualized in the 3D plot but crucial to highlight given the central role of this morphological trait in the study. This reinforces the relevance of considering PC4 when interpreting the influence of FHW position on behavioral and thermal responses.

These findings underlined the potential for these markers to be used as non-invasive indicators of temperament or physiological sensitivity. Notably, Górecka et al. (27) linked FHW characteristics to horses’ responses in novel object and handling tests, providing empirical support for the predictive value of these external features.

This differentiation reinforces the idea that behavior and physiological responses are not random but organized into definable profiles that can be partially predicted from physical traits like FHW location. Although FHW alone did not unequivocally predict reactivity or behavior, their integration with behavioral and physiological data helped revealed complex functional profiles that were valuable in veterinary practice, breeding, and sports. This approach, supported by Bartolomé and Cockram (35), Visser et al. (25), and Grandin and Deesing (17), suggested a more comprehensive horse evaluation that considered interactions between stable morphological traits and dynamic emotional responses.

Hence, given the relative ease of assessing FHW, future research should expand to larger, more diverse populations and include genetic, developmental, and behavioral metrics to understand the biological underpinnings of these associations (37).

Some limitations of the present study should be acknowledged. First, the population was restricted to gray-coated male PRE horses participating in Dressage finals, which may limit the generalization of the results to other sexes, coat colors, breeds, or equestrian disciplines. Second, behavioral traits were assessed through questionnaires completed by the regular rider or caretaker, which, although commonly used and informative in large-scale studies, may involve a degree of subjectivity. Finally, while the integrative approach combining morphological, behavioral, and physiological variables allowed the identification of functional profiles, causal relationships cannot be inferred and should be explored in future studies incorporating longitudinal designs or additional neurophysiological indicators.

## 5 Conclusion

This study revealed that facial hair whorls located at eye level were predominant in the evaluated PRE horses and were associated with heightened post-competition ocular temperatures, suggesting greater physiological stress reactivity. Behavioral evaluations identified a desirable, balanced temperament for Dressage, with strong interrelations among traits like *cooperation*, *endurance*, and *learning ability*. Ocular thermography proved to be a reliable, non-invasive tool to assess emotional reactivity, with temperature fluctuations closely linked to behavioral traits such as *nervousness* and *excitability*. Horses with multiple eye-level FHW exhibited higher stress responses and tended to show lower *cooperation* scores, suggesting potential cumulative phenotypic effects. When integrating all variables, multivariate analysis identified three functional horse profiles (functional, reactive and intermediate), reinforcing the value of integrating morphological, physiological, and behavioral data to support selection and training strategies in Dressage horses. From an applied perspective, facial hair whorl patterns should not be considered predictive tools on their own, but rather as complementary, easily observable markers that, when integrated with behavioral and physiological assessments, may support early profiling, management decisions, and training strategies in Dressage horses.

## Data Availability

The datasets presented in this study can be found in online repositories. The names of the repository/repositories and accession number(s) can be found below: https://doi.org/10.5281/zenodo.17158466.
